# Crystal structure of a 2:1 co-crystal of the anti­cancer drug lenalidomide with 1,3-resorcinol

**DOI:** 10.1107/S205698902601114X

**Published:** 2026-10-30

**Authors:** Sejal Kaldoke, Niteen B. Dabke, Nagabhushan Shet, Rajesh G. Gonnade

**Affiliations:** a Physical and Materials Chemistry Division CSIR-National Chemical Laboratory, Dr Homi Bhabha Road Pashan Pune Maharashtra 411008 India; b Academy of Scientific and Innovative Research (AcSIR) Ghaziabad 201002 India; Indian Institute of Science Education and Research Bhopal, India

**Keywords:** crystal structure, anti­cancer, lenalidomide, hydrogen bond, π-stacking

## Abstract

A 2:1 cocrystal of lenalidomide and 1,3-resorcinol [systematic name: 3-(4-amino-1-oxo-1,3-di­hydro-2*H*-isoindol-2-yl)piperidine-2,6-dione–benzene-1,3-diol(2/1), 2C_13_H_13_N_3_O_3_·C_6_H_6_O_2_] was synthesized, and its crystal structure was elucidated by single-crystal X-ray diffraction (SCXRD). The cocrystal crystallizes in the triclinic space group *P*1. The two symmetry-independent lenalidomide mol­ecules form a hydrogen-bonded dimeric assembly through N—H⋯O inter­actions, generating an *R*_2_^2^(8) ring motif. In addition, O—H⋯O hydrogen bonds involving the hydroxyl groups of 1,3-resorcinol and the carbonyl oxygen atoms of the isoindolinone and glutarimide moieties of lenalidomide further contribute to the cohesion and organization of the crystal packing. The crystal structure is further consolidated by several close π–π stacking inter­actions involving the π-electrons of the isoindolinone moiety. Hirshfeld surface analysis and the corresponding two-dimensional fingerprint plots were employed to identify and qu­antify the principal inter­molecular contacts contributing to the cohesion of the cocrystal structure.

## Abstract 2

The cocrystal of the anti­cancer drug lenalidomide with 1,3-resorcinol crystallizes in the triclinic centrosymmetric *P*1 space group, with two mol­ecules of lenalidomide and one mol­ecule of 1,3-resorcinol present in the asymmetric unit. The crystal structure is primarily consolidated by a network of strong N—H⋯O hydrogen bonds involving the glutarimide moieties of the lenalidomide mol­ecule. In addition, O—H⋯O hydrogen bonds involving the hydroxyl groups of 1,3-resorcinol and the carbonyl oxygen atoms of the isoindolinone and glutarimide moieties of lenalidomide further contribute to the cohesion and organization of the crystal packing.

## 1. Chemical context

Lenalidomide (**LEN**), a synthetic thalidomide analogue, is an immunomodulatory drug used in the treatment of multiple myeloma and myelodysplastic syndromes associated with deletion of chromosome 5q [del(5q)] (Richardson *et al.*, 2002 ▸; List *et al.*, 2006 ▸). Reported pharmacological effects include immunomodulatory and anti­angiogenic activity (Kotla *et al.*, 2009 ▸). Also in multiple myeloma, lenalidomide has also been shown to promote cereblon-dependent degradation of IKZF1 and IKZF3 (Krönke *et al.*, 2014 ▸). The solid-state landscape of **LEN** includes anhydrous and hydrated forms, polymorphs and solvates (Ravikumar & Sridhar, 2009 ▸; Chennuru *et al.*, 2017 ▸; Jia *et al.*, 2019 ▸). Cocrystals of **LEN** with urea and 3,5-di­hydroxy­benzoic acid, gallic acid and nicotinamide have been reported, with selected systems showing changes in apparent solubility or dissolution under the conditions studied (Song *et al.*, 2014 ▸, Song *et al.*, 2015 ▸; Wang *et al.*, 2022 ▸). A crystalline form of lenalidomide (**LEN**) with resorcinol (**RES**) was previously disclosed in patent US 12098136 B2, accompanied by DSC, PXRD and NMR data; however, its crystal structure was not reported (Bhat *et al.*, 2024 ▸). Here, we report the single-crystal X-ray structure of the **LEN:RES** cocrystal (2:1), providing a crystallographic description of its mol­ecular arrangement and inter­molecular inter­actions.
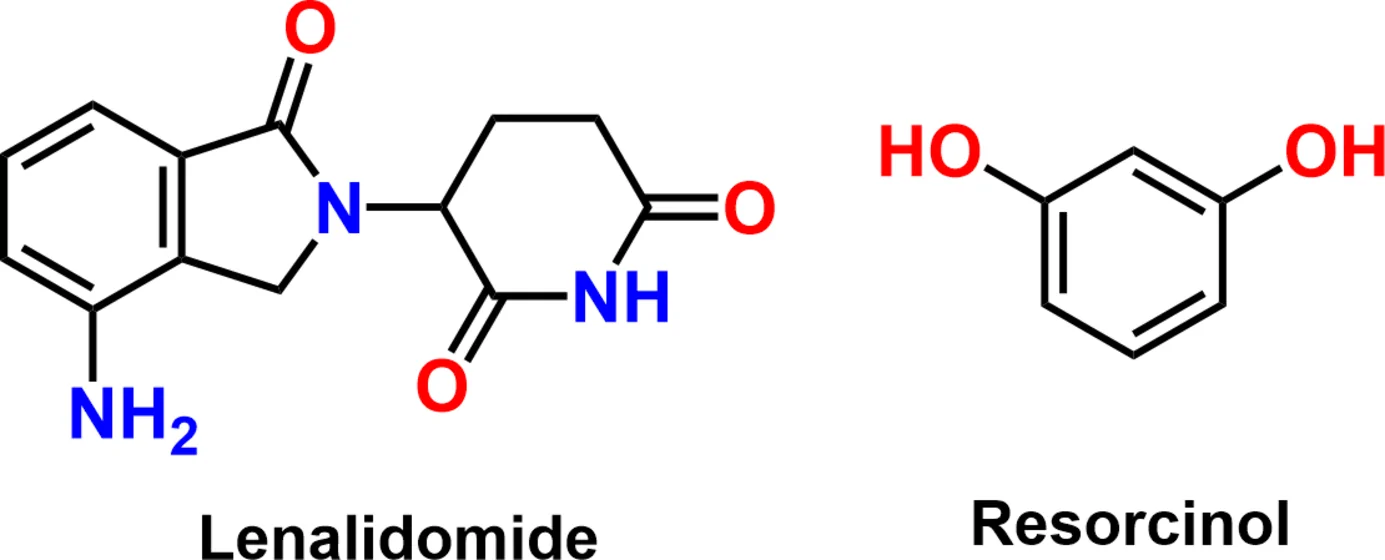


## 2. Structural commentary

**Figure 1:**
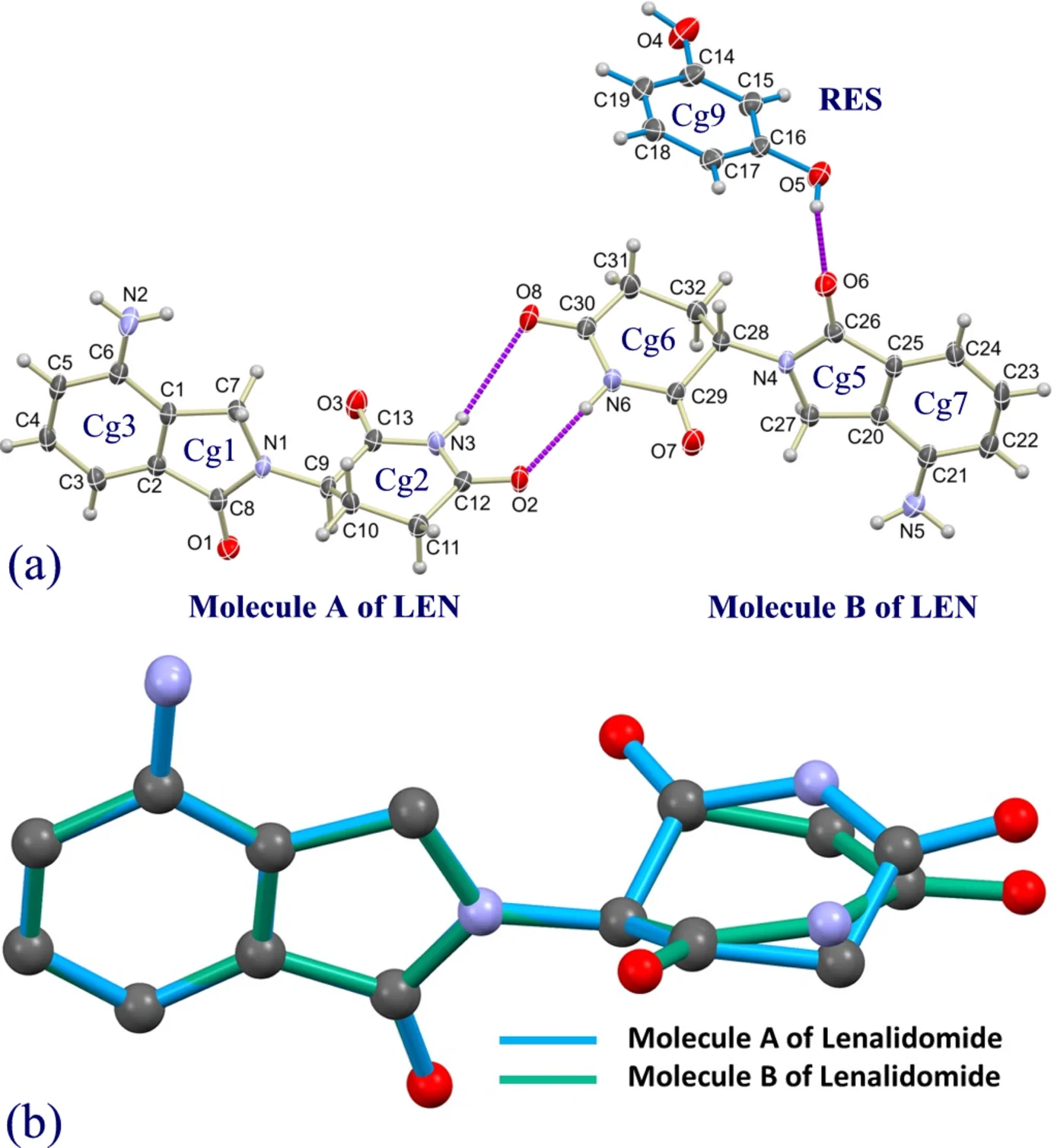
(*a*) The **LEN:RES** cocrystal with thermal displacement ellipsoids drawn at the 30% probability level. Atom labelling is provided for identification; hydrogen atoms are depicted as spheres of arbitrary radii for clarity. Hydrogen-bonding inter­actions are shown as violet dashed lines. (*b*) Overlay of two symmetry-independent mol­ecules of **LEN** within the asymmetric unit of the **LEN:RES** cocrystal.

The crystal structure of the **LEN:RES** (2:1) cocrystal was unambiguously established by single-crystal X-ray diffraction (SCXRD) analysis. The cocrystal crystallizes in the triclinic crystal system with the centrosymmetric space group *P*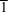 (space group No. 2). The asymmetric unit comprises two independent mol­ecules of **LEN** and one mol­ecule of **RES**, corresponding to a 2:1 stoichiometric ratio (Fig. 1 ▸*a*). The two crystallographically independent **LEN** mol­ecules are designated *A* and *B*. A structural overlay of mol­ecules *A* and *B* reveals a distinct conformational difference in the glutarimide ring (Fig. 1 ▸*b*). The dihedral angle between a plane between the isoindolinone-derived aromatic system and the glutarimide ring is 86.93 (3)° for mol­ecule *A* and 78.96 (3)° for mol­ecule *B*. Thus, mol­ecule *A* adopts a more orthogonal, and consequently less twisted, conformation with respect to the defined ring planes than mol­ecule *B*. The mol­ecules present in the asymmetric unit are inter­connected through a network of hydrogen-bonding inter­actions. The two crystallographically independent **LEN** mol­ecules, *A* and *B*, are connected through N—H⋯O hydrogen bonds, generating a cyclic 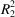
(8) ring motif. In addition, the **RES** mol­ecule participates in O—H⋯O hydrogen bonding engaging its hydroxyl group with the carbonyl oxygen atom of adjacent **LEN** mol­ecule *B*, thereby further linking the mol­ecular components within the asymmetric unit.

## 3. Supra­molecular features

**Figure 2:**
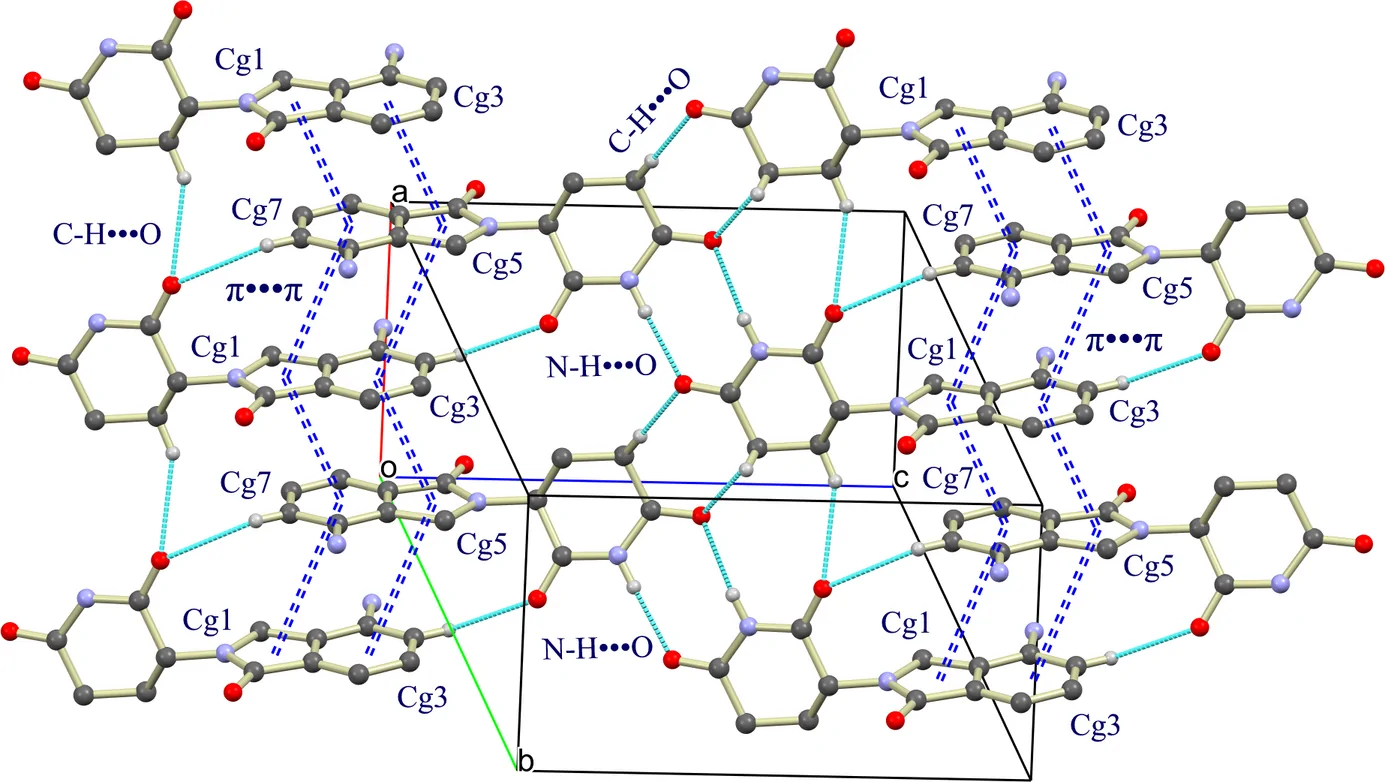
View of the mol­ecular packing along the *b* axis in the **LEN:RES** cocrystal, showing the inter­molecular C—H⋯O and N—H⋯O hydrogen bonds and π–π stacking inter­actions.

**Figure 3:**
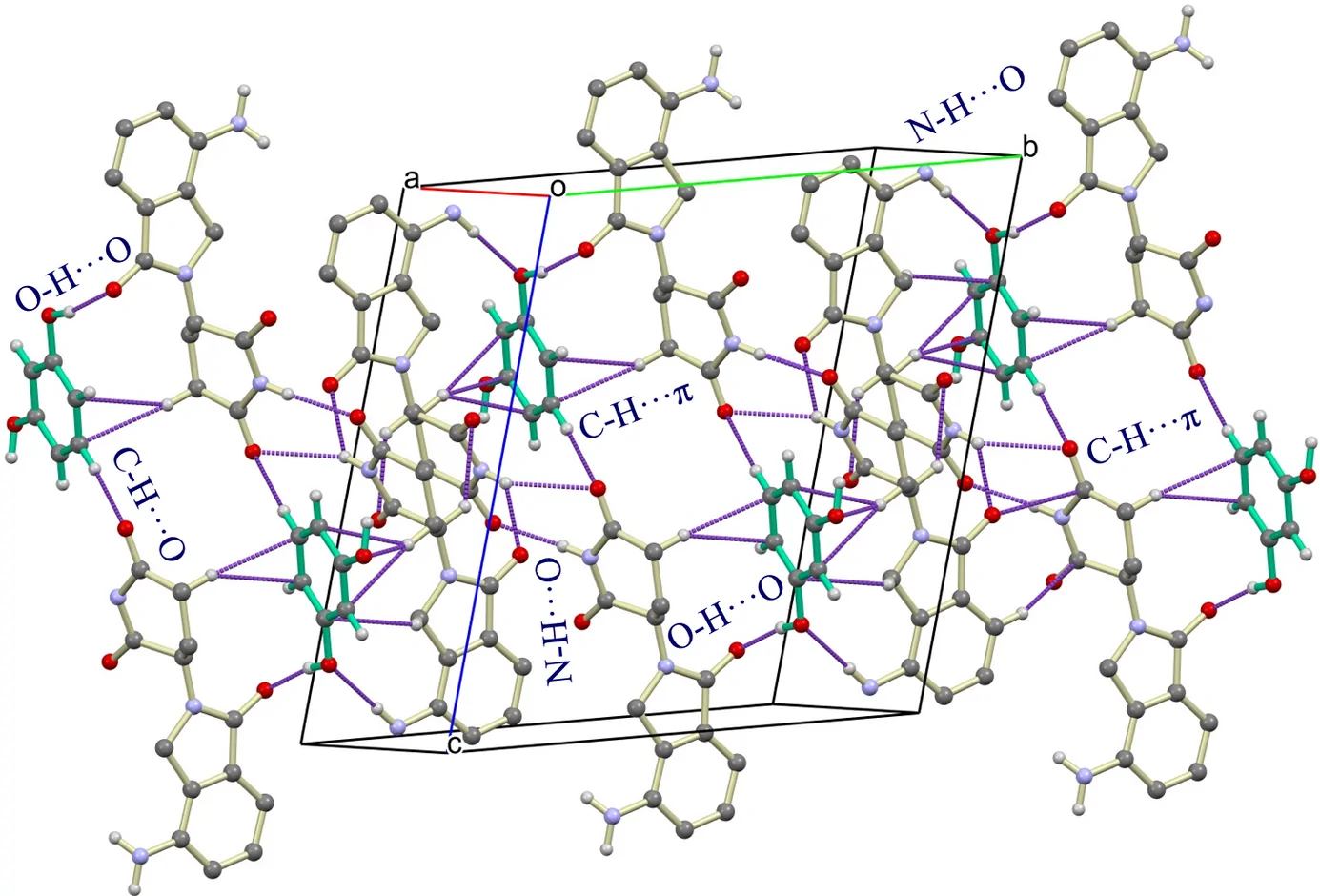
View of the mol­ecular packing along the *a* axis in the **LEN:RES** cocrystal, joined using C—H⋯O, N—H⋯O, O—H⋯O and C—H⋯π inter­actions.

The mol­ecular packing viewed along the *b* axis reveals that the **LEN** mol­ecules are arranged along the *a*-axis direction through short π–π stacking inter­actions involving the isoindolinone moiety, with centroid-to-centroid distances ranging from 3.5410 (15) to 4.0014 (17) Å (see entries 14-25 of Table 1 ▸). These inter­actions generate a stacked one-dimensional supra­molecular assembly extending along the *a*-axis direction. The stacked assemblies are further connected along the *c*-axis direction through dimeric C—H⋯O and N—H⋯O hydrogen-bonding inter­actions (C31—H31*B*⋯O2^vii^, N3—H3⋯O8, and N6—H6⋯O2; Table 1 ▸), both forming cyclic 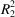
(8) ring motifs. Consequently, an extended and compact supra­molecular architecture is generated in the *ac* plane (Fig. 2 ▸). The packing arrangement viewed along the *a*-axis shows that the **RES** mol­ecule is positioned between two **LEN** mol­ecules and is involved in N—H⋯O (N2—H2*B*⋯O5^ii^ and N3—H3⋯O1^iv^), O—H⋯O (O5—H5*A*⋯O6) and C—H⋯π (C10—H10A⋯*Cg*9^ii^) inter­actions. In particular, C—H⋯π inter­actions occur between the hydrogen atoms associated with the glutarimide moiety of **LEN** and the π-electron cloud of the aromatic ring of **RES**, contributing to the formation of a one-dimensional supra­molecular assembly along the *b*-axis direction. In addition, in the packing along the *c*-axis direction, **RES** inter­acts with adjacent **LEN** mol­ecules through C—H⋯O contacts involving hydrogen atoms of **RES** and the carbonyl oxygen atoms of the glutarimide moieties of **LEN** (Fig. 3 ▸). These inter­molecular inter­actions collectively consolidate the three-dimensional crystal packing.

## 4. Hirshfeld surface analysis

**Figure 4:**
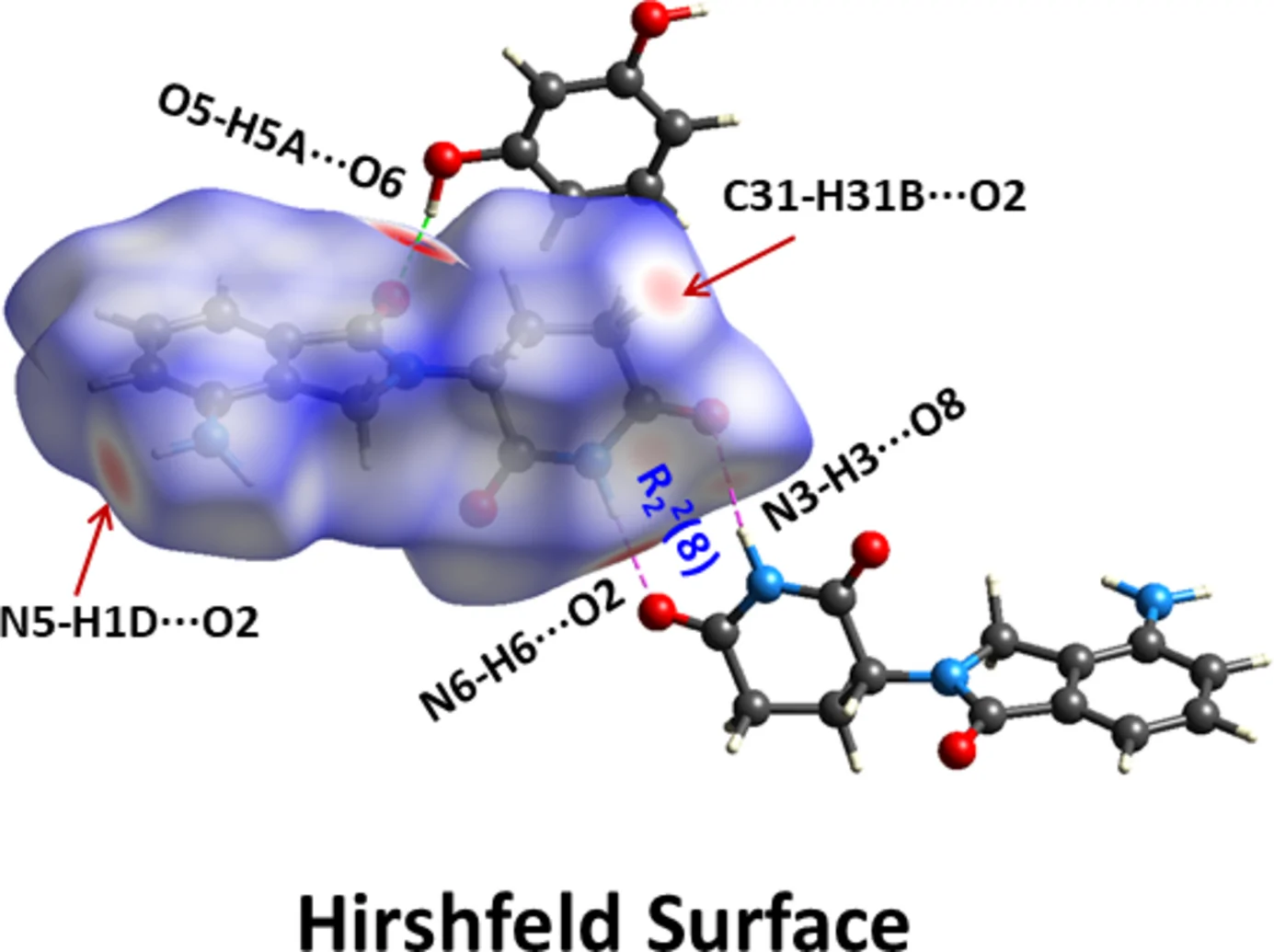
Visualization of the Hirshfeld surface of mol­ecule B of **LEN**, within the **LEN:RES** cocrystal.

**Figure 5:**
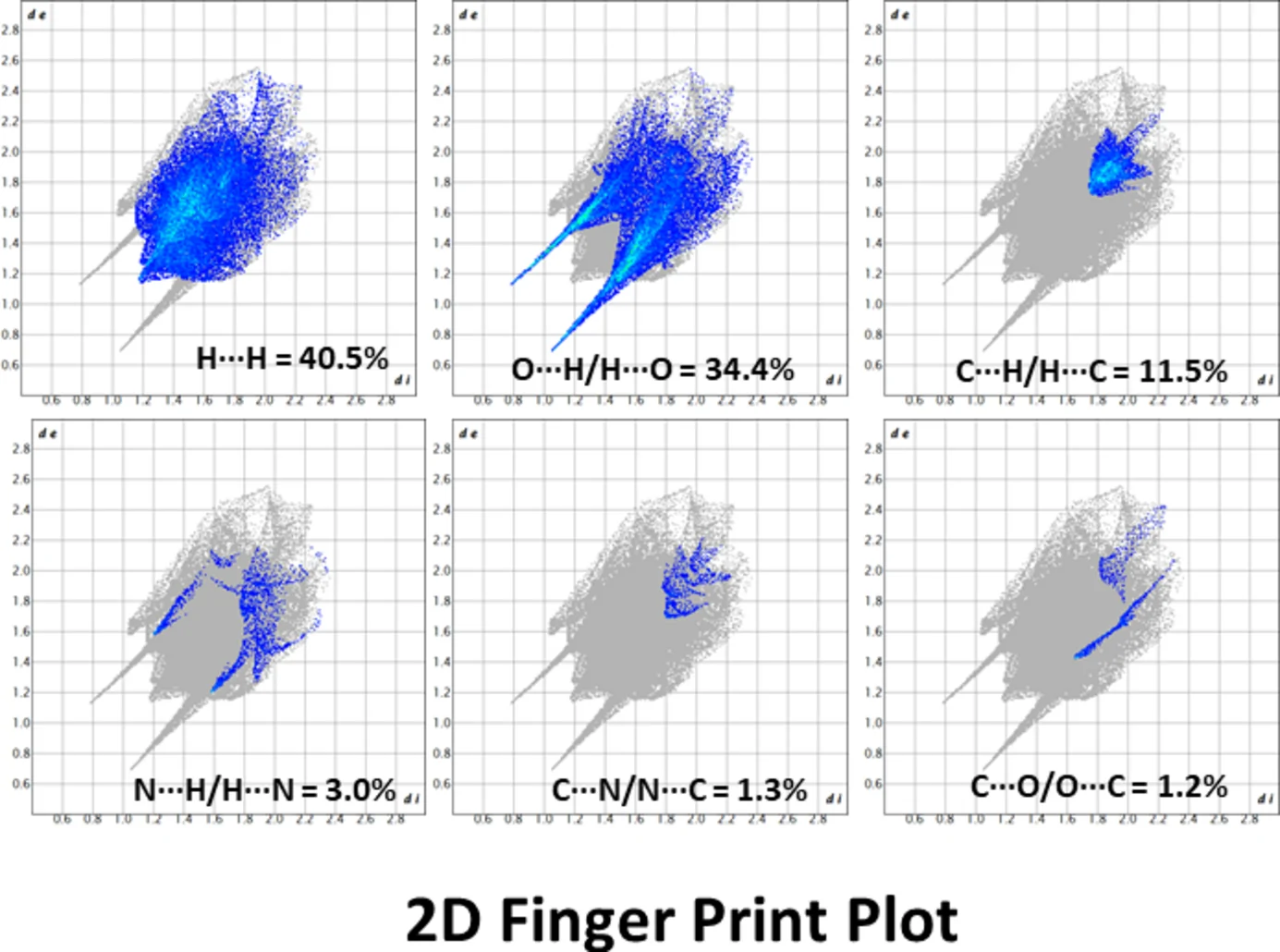
Two-dimensional fingerprint plot of mol­ecule *B* of **LEN** in the **LEN:RES** cocrystal, showing the percentage contributions of different inter­molecular inter­actions to the Hirshfeld surface area.

Hirshfeld surface (HS) analysis (Spackman & Jayatilaka, 2009 ▸) and two-dimensional fingerprint plots (Spackman & McKinnon, 2002 ▸) were performed using *CrystalExplorer* (Spackman *et al.*, 2021 ▸). The Hirshfeld surface mapped over *d*_norm_ for mol­ecule *B* of **LEN** in the cocrystal reveals several prominent red spots, indicating close contacts between **LEN** and **RES**. The Hirshfeld surface analysis was performed only for molecule *B* of **LEN** because it participates in O—H⋯O hydrogen bonding with the **RES** coformer, and N—H⋯O hydrogen bonding with molecule *A* of **LEN**, forming a dimeric motif. One of the most prominent red spots corresponds to the strong O—H⋯O hydrogen-bonding inter­action between the hydroxyl group of **RES** and the carbonyl oxygen atom of **LEN**. The other prominent red spots are associated with N—H⋯O hydrogen-bonding inter­actions, which generate an 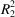
(8) hydrogen-bonded ring motif (Fig. 4 ▸). The two-dimensional fingerprint plots further illustrate the principal inter­molecular contacts contributing to the consolidation of the cocrystal structure. In particular, the H⋯H and O⋯H/H⋯O contacts exhibit prominent features at (*d*_i_ + *d*_e_) values of approximately 2.4 and 2.0 Å, respectively (Fig. 5 ▸). These values are shorter than the corresponding sums of the van der Waals radii, indicating the presence of significant close inter­molecular contacts.

## 5. Database survey

A survey of the Cambridge Structural Database (CSD, Version 6.01, Groom *et al.*, 2016 ▸) using ConQuest (Bruno *et al.*, 2002 ▸) identified 16 crystal structures based on **LEN**. Among these, two structures correspond to polymorphs of **LEN** (AJISES; Ravikumar & Sridhar, 2009 ▸, AJISES01; Chennuru *et al.*, 2017 ▸), while two are hydrate forms (AJISIW; Ravikumar & Sridhar, 2009 ▸, ICOSUR; Chennuru *et al.*, 2017 ▸). Solvate structures containing DMF (ICOTEC; Chennuru *et al.*, 2017 ▸), acetone (ICOTIG; Chennuru *et al.*, 2017 ▸), DMSO (ICOTOM; Chennuru *et al.*, 2017 ▸), and aceto­nitrile (UZULUZ) have also been reported. In addition, **LEN** cocrystals and cocrystal hydrates have been reported with gallic acid (HUVGAI and HUVFUB; Song *et al.*, 2015 ▸), 3,5-di­hydroxy­benzoic acid (XODPIR and XODPEN; Song *et al.*, 2014 ▸), urea (XODPOX; Song *et al.*, 2014 ▸), melamine (NUNSAU; Screen *et al.*, 2025 ▸) and acesulfame (ZIDQUB and ZIDQUB01; Chen *et al.*, 2019 ▸). Inter­estingly, one of the reported crystal structures contains protonated **LEN**, with a chloride ion (Cl^−^) serving as the counter-ion to maintain charge neutrality (VAGMEZ; Valdivia-Berroeta *et al.*, 2021 ▸). However, to the best of our knowledge, no crystal structure of a **LEN:RES** has been reported to date.

## 6. Synthesis and crystallization

The **LEN:RES** cocrystal was prepared by a combination of liquid-assisted grinding and slow-evaporation crystallization. **LEN** (60 mg, 0.231 mmol) and **RES** (25.5 mg, 0.231 mmol) were taken in a 1:1 molar ratio and manually ground together in a mortar and pestle for approximately 10 min in the presence of a few drops of a 1:1 (*v*/*v*) acetone-ethanol solvent mixture. The resulting solid was subsequently dissolved in 1:1:1:1 (*v*/*v*/*v*/*v*) mixture of isopropyl alcohol, methanol, aceto­nitrile, and water and gently heated to ensure complete dissolution. The resulting solution was filtered to remove any undissolved material and transferred to a crystallization vessel. The solvent was allowed to evaporate slowly at room temperature (298 K). After 4–5 days, yellow, plate-like crystals suitable for single-crystal X-ray diffraction analysis were obtained.

## 7. Refinement

Crystal data, data collection and structure refinement details are summarized in Table 2 ▸. All H atoms were placed in geometrically idealized positions. The H atoms attached to O4 and O5 were located in difference-Fourier maps and refined isotropically. The H atom attached to N3 was placed in a geometrically idealized position and the N3—H3 bond length was restrained to 0.95 Å using a DFIX restraint.

## Supplementary Material

Crystal structure: contains datablock(s) I. DOI: 10.1107/S205698902601114X/dx2074sup1.cif

Structure factors: contains datablock(s) I. DOI: 10.1107/S205698902601114X/dx2074Isup3.hkl

Supporting information file. DOI: 10.1107/S205698902601114X/dx2074Isup3.cml

CCDC reference: 2598460

Additional supporting information:  crystallographic information; 3D view; checkCIF report

## Figures and Tables

**Table 1:** Hydrogen-bond geometry (Å, °) Ring centroids are defined as: *Cg*1 = N1/C7/C1/C2/C8; *Cg*2 = N3/C9–C13; *Cg*3 = C1–C6;*Cg*5 = N4/C20/C25–C27; *Cg*6 = N6/C28–C32; *Cg*7 = C20–C25; and *Cg*9 = C14–C19. *Cg*⋯*Cg* denotes the centroid—centroid distance associated with π–π inter­actions.

*D*—H⋯*A*	*D*—H	H⋯*A*	*D*⋯*A*	*D*—H⋯*A*
O4—H1⋯O8^i^	0.93 (3)	1.92 (3)	2.816 (3)	161 (3)
O5—H5*A*⋯O6	0.85 (3)	1.87 (3)	2.710 (3)	171 (3)
N2—H2*B*⋯O5^ii^	0.78 (3)	2.56 (3)	3.310 (3)	163 (3)
N2—H2*A*⋯O5^iii^	0.83 (3)	2.28 (3)	3.015 (3)	148 (3)
N3—H3⋯O1^iv^	0.89 (2)	2.31 (2)	3.022 (3)	137 (2)
N3—H3⋯O8	0.89 (2)	2.51 (2)	3.256 (3)	141 (2)
N5—H1*D*⋯O2^v^	0.92 (3)	2.23 (3)	3.144 (3)	172 (2)
N6—H6⋯O2	0.92 (3)	2.00 (3)	2.915 (3)	171 (2)
C9—H9⋯O3^iv^	0.98	2.65	3.409 (3)	135
C17—H17⋯O4^vi^	0.93	2.57	3.263 (3)	131
C27—H27*B*⋯O7	0.97	2.58	3.106 (3)	114
C31—H31*B*⋯O2^vii^	0.97	2.47	3.440 (3)	178
C10—H10*A*⋯*Cg*9^ii^		2.65	3.581 (3)	161
*Cg*1⋯*Cg*7^iii^			3.7109 (16)	
*Cg*1⋯*Cg*7^viii^			4.0014 (17)	
*Cg*3⋯*Cg*5^iii^			3.7997 (17)	
*Cg*3⋯*Cg*5^viii^			3.9603 (17)	
*Cg*3⋯*Cg*7^iii^			3.6971 (17)	
*Cg*3⋯*Cg*7^viii^			3.6644 (17)	
*Cg*3⋯*Cg*8^iii^			3.5854 (15)	
*Cg*3⋯*Cg*8^viii^			3.6382 (15)	
*Cg*4⋯*Cg*7^iii^			3.5410 (15)	
*Cg*4⋯*Cg*7^viii^			3.6602 (15)	
*Cg*4⋯*Cg*8^iii^			3.6763 (13)	
*Cg*4⋯*Cg*8^viii^			3.8723 (13)	

Symmetry codes: (i) 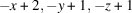; (ii) 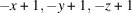; (iii) 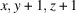; (iv) 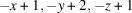; (v) 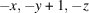; (vi) 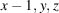; (vii) 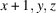; (viii) 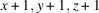.

**Table 2:** Experimental details

Crystal data	
Chemical formula	2C_13_H_13_N_3_O_3_·C_6_H_6_O_2_
*M*_r_	628.63
Crystal system, space group	Triclinic, *P* 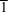
Temperature (K)	297
*a*, *b*, *c* (Å)	7.0508 (3), 14.1141 (6), 16.3951 (9)
α, β, γ (°)	110.596 (2), 100.956 (2), 101.880 (2)
*V* (Å^3^)	1431.91 (12)
*Z*	2
Radiation type	Mo *K*α
μ (mm^−1^)	0.11
Crystal size (mm)	0.14 × 0.09 × 0.04

Data collection	
Diffractometer	Bruker D8 VENTURE Kappa Duo PHOTON II CPAD
Absorption correction	Multi-scan (*SADABS*; Krause *et al.*, 2015 ▸)
*T*_min_, *T*_max_	0.632, 0.746
No. of measured, independent and observed [*I* > 2σ(*I*)] reflections	44385, 7160, 3351
*R*_int_	0.138
(sin θ/λ)_max_ (Å^−1^)	0.671

Refinement	
*R*[*F*^2^ > 2σ(*F*^2^)], *wR*(*F*^2^), *S*	0.061, 0.168, 0.99
No. of reflections	7160
No. of parameters	439
No. of restraints	1
H-atom treatment	H atoms treated by a mixture of independent and constrained refinement
Δρ_max_, Δρ_min_ (e Å^−3^)	0.30, −0.26

Computer programs: *APEX3* and *SAINT* (Bruker, 2016 ▸), *SHELXS97* (Sheldrick, 2008 ▸), *SHELXL2014/7* (Sheldrick, 2015 ▸), *ORTEP-3 for Windows* (Farrugia, 2012 ▸), *Mercury* (Macrae *et al.*, 2020 ▸), *SHELXTL* (Sheldrick, 2008 ▸), *PLATON* (Spek, 2020 ▸) and *publCIF* (Westrip, 2010 ▸).

## supplementary crystallographic information

### 3-(4-Amino-1-oxo-1,3-dihydro-2H-isoindol-2-yl)piperidine-2,6-dione–benzene-1,3-diol (2/1) Crystal data

**Table:**

2C_13_H_13_N_3_O_3_·C_6_H_6_O_2_	*Z* = 2
*M**_r_* = 628.63	*F*(000) = 660
Triclinic, *P*1	*D*_x_ = 1.458 Mg m^−^^3^
*a* = 7.0508 (3) Å	Mo *K*α radiation, λ = 0.71073 Å
*b* = 14.1141 (6) Å	Cell parameters from 7164 reflections
*c* = 16.3951 (9) Å	θ = 2.5–28.5°
α = 110.596 (2)°	µ = 0.11 mm^−^^1^
β = 100.956 (2)°	*T* = 297 K
γ = 101.880 (2)°	Plate, pale yellow
*V* = 1431.91 (12) Å^3^	0.14 × 0.09 × 0.04 mm

### 3-(4-Amino-1-oxo-1,3-dihydro-2H-isoindol-2-yl)piperidine-2,6-dione–benzene-1,3-diol (2/1) Data collection

**Table:**

Bruker D8 VENTURE Kappa Duo PHOTON II CPAD diffractometer	7160 independent reflections
Radiation source: micro-focus sealed tube, Incoatech IµS HB	3351 reflections with *I* > 2σ(*I*)
Multilayer mirrors monochromator	*R*_int_ = 0.138
φ and ω scans	θ_max_ = 28.5°, θ_min_ = 2.5°
Absorption correction: multi-scan (SADABS; Krause *et al.*, 2015)	*h* = −9→9
*T*_min_ = 0.632, *T*_max_ = 0.746	*k* = −18→18
44385 measured reflections	*l* = −21→21

### 3-(4-Amino-1-oxo-1,3-dihydro-2H-isoindol-2-yl)piperidine-2,6-dione–benzene-1,3-diol (2/1) Refinement

**Table:**

Refinement on *F*^2^	1 restraint
Least-squares matrix: full	Hydrogen site location: mixed
*R*[*F*^2^ > 2σ(*F*^2^)] = 0.061	H atoms treated by a mixture of independent and constrained refinement
*wR*(*F*^2^) = 0.168	*w* = 1/[σ^2^(*F*_o_^2^) + (0.069*P*)^2^] where *P* = (*F*_o_^2^ + 2*F*_c_^2^)/3
*S* = 0.99	(Δ/σ)_max_ = 0.001
7160 reflections	Δρ_max_ = 0.30 e Å^−^^3^
439 parameters	Δρ_min_ = −0.26 e Å^−^^3^

### 3-(4-Amino-1-oxo-1,3-dihydro-2H-isoindol-2-yl)piperidine-2,6-dione–benzene-1,3-diol (2/1) Special details

**Table:**

Geometry. All esds (except the esd in the dihedral angle between two l.s. planes) are estimated using the full covariance matrix. The cell esds are taken into account individually in the estimation of esds in distances, angles and torsion angles; correlations between esds in cell parameters are only used when they are defined by crystal symmetry. An approximate (isotropic) treatment of cell esds is used for estimating esds involving l.s. planes.

### 3-(4-Amino-1-oxo-1,3-dihydro-2H-isoindol-2-yl)piperidine-2,6-dione–benzene-1,3-diol (2/1) Fractional atomic coordinates and isotropic or equivalent isotropic displacement parameters (Å^2^)

**Table:**

	*x*	*y*	*z*	*U*_iso_*/*U*_eq_	
O1	0.4392 (3)	1.21869 (13)	0.66753 (11)	0.0466 (5)	
O2	0.1038 (3)	0.70701 (13)	0.37776 (11)	0.0471 (5)	
O3	0.6444 (3)	0.96443 (14)	0.58963 (12)	0.0526 (5)	
N1	0.4203 (3)	1.06732 (14)	0.69316 (12)	0.0335 (5)	
N2	0.6294 (4)	1.08409 (19)	0.96980 (16)	0.0551 (7)	
N3	0.3680 (3)	0.83778 (15)	0.48570 (14)	0.0373 (5)	
C1	0.5573 (4)	1.14192 (17)	0.84840 (15)	0.0322 (6)	
C2	0.5571 (3)	1.22231 (18)	0.81917 (16)	0.0331 (6)	
C3	0.6287 (4)	1.32822 (18)	0.87877 (17)	0.0402 (6)	
H3A	0.628058	1.382037	0.858556	0.048*	
C4	0.7014 (4)	1.34981 (19)	0.97012 (17)	0.0442 (7)	
H4	0.750506	1.419965	1.012339	0.053*	
C5	0.7028 (4)	1.27023 (19)	0.99993 (16)	0.0401 (6)	
H5	0.753498	1.288036	1.061774	0.048*	
C6	0.6302 (4)	1.16338 (19)	0.93995 (16)	0.0356 (6)	
C7	0.4697 (4)	1.03661 (17)	0.76899 (15)	0.0340 (6)	
H7A	0.567555	0.997243	0.762260	0.041*	
H7B	0.349737	0.994421	0.774725	0.041*	
C8	0.4685 (4)	1.17463 (18)	0.71944 (16)	0.0341 (6)	
C9	0.3236 (4)	0.99346 (18)	0.60035 (15)	0.0335 (6)	
H9	0.299217	1.035799	0.565413	0.040*	
C10	0.1187 (4)	0.91915 (19)	0.58530 (16)	0.0385 (6)	
H10A	0.134266	0.876290	0.619736	0.046*	
H10B	0.029641	0.960111	0.606562	0.046*	
C11	0.0268 (4)	0.84802 (19)	0.48517 (17)	0.0416 (6)	
H11A	−0.097317	0.796450	0.477254	0.050*	
H11B	−0.007630	0.890553	0.452601	0.050*	
C12	0.1649 (4)	0.79088 (19)	0.44478 (16)	0.0346 (6)	
C13	0.4619 (4)	0.93194 (18)	0.56100 (16)	0.0345 (6)	
H3	0.443 (4)	0.8010 (18)	0.4577 (16)	0.052*	
H2A	0.670 (4)	1.100 (2)	1.0250 (19)	0.052*	
H2B	0.592 (4)	1.026 (2)	0.9329 (18)	0.052*	
O4	1.2550 (3)	0.3382 (2)	0.38207 (14)	0.0767 (7)	
O5	0.6176 (3)	0.16702 (16)	0.16464 (13)	0.0563 (6)	
C14	1.0483 (4)	0.3148 (2)	0.36367 (19)	0.0481 (7)	
C15	0.9331 (4)	0.2529 (2)	0.27456 (18)	0.0470 (7)	
H15	0.995148	0.228355	0.229379	0.056*	
C16	0.7265 (4)	0.22825 (19)	0.25380 (17)	0.0400 (6)	
C17	0.6315 (4)	0.2623 (2)	0.32043 (18)	0.0447 (7)	
H17	0.491186	0.243909	0.306246	0.054*	
C18	0.7511 (5)	0.3246 (2)	0.40876 (18)	0.0484 (7)	
H18	0.689303	0.348195	0.454229	0.058*	
C19	0.9582 (5)	0.3523 (2)	0.43099 (17)	0.0469 (7)	
H19	1.036325	0.395666	0.490416	0.056*	
H1	1.308 (5)	0.359 (2)	0.444 (2)	0.070*	
H5A	0.494 (5)	0.166 (2)	0.152 (2)	0.070*	
O6	0.2332 (3)	0.17291 (13)	0.11279 (11)	0.0478 (5)	
O7	0.0550 (3)	0.46102 (15)	0.18180 (13)	0.0553 (5)	
O8	0.5573 (3)	0.63971 (15)	0.44179 (12)	0.0543 (5)	
N4	0.2641 (3)	0.32938 (14)	0.09561 (13)	0.0360 (5)	
N5	0.1058 (4)	0.3344 (2)	−0.17610 (17)	0.0524 (7)	
N6	0.3138 (3)	0.55180 (16)	0.30871 (14)	0.0415 (6)	
C20	0.1513 (4)	0.26472 (18)	−0.06018 (16)	0.0337 (6)	
C21	0.0881 (4)	0.24901 (19)	−0.15121 (16)	0.0375 (6)	
C22	0.0140 (4)	0.1445 (2)	−0.21483 (17)	0.0454 (7)	
H22	−0.029956	0.130577	−0.276211	0.054*	
C23	0.0040 (4)	0.0602 (2)	−0.18903 (18)	0.0482 (7)	
H23	−0.047229	−0.008665	−0.233698	0.058*	
C24	0.0678 (4)	0.07543 (19)	−0.09901 (17)	0.0434 (7)	
H24	0.062888	0.019145	−0.081646	0.052*	
C25	0.1398 (4)	0.18046 (18)	−0.03610 (16)	0.0345 (6)	
C26	0.2149 (4)	0.22211 (18)	0.06352 (17)	0.0351 (6)	
C27	0.2335 (4)	0.36639 (18)	0.02291 (15)	0.0369 (6)	
H27A	0.359950	0.409290	0.022747	0.044*	
H27B	0.137622	0.407037	0.028206	0.044*	
C28	0.3475 (4)	0.39755 (18)	0.19125 (16)	0.0352 (6)	
H28	0.340619	0.351277	0.223981	0.042*	
C29	0.2221 (4)	0.47085 (19)	0.22422 (16)	0.0358 (6)	
C30	0.5058 (4)	0.57531 (18)	0.36249 (17)	0.0389 (6)	
C31	0.6447 (4)	0.5217 (2)	0.32072 (17)	0.0452 (7)	
H31A	0.670477	0.472816	0.347679	0.054*	
H31B	0.772645	0.574642	0.335705	0.054*	
C32	0.5682 (4)	0.46140 (19)	0.21851 (17)	0.0419 (6)	
H32A	0.648126	0.414196	0.198385	0.050*	
H32B	0.581853	0.510698	0.189556	0.050*	
H1C	0.079 (5)	0.388 (2)	−0.1394 (19)	0.063*	
H1D	0.046 (4)	0.315 (2)	−0.2365 (19)	0.063*	
H6	0.241 (4)	0.596 (2)	0.3330 (19)	0.063*	

### 3-(4-Amino-1-oxo-1,3-dihydro-2H-isoindol-2-yl)piperidine-2,6-dione–benzene-1,3-diol (2/1) Atomic displacement parameters (Å^2^)

**Table:**

	*U*^11^	*U*^22^	*U*^33^	*U*^12^	*U*^13^	*U*^23^
O1	0.0613 (13)	0.0413 (10)	0.0367 (11)	0.0153 (9)	0.0107 (10)	0.0169 (9)
O2	0.0483 (12)	0.0406 (10)	0.0319 (10)	0.0099 (9)	0.0041 (9)	−0.0029 (9)
O3	0.0350 (12)	0.0613 (12)	0.0403 (11)	0.0071 (10)	0.0090 (10)	0.0015 (10)
N1	0.0424 (13)	0.0300 (10)	0.0210 (11)	0.0069 (9)	0.0071 (10)	0.0054 (9)
N2	0.083 (2)	0.0437 (14)	0.0260 (14)	0.0064 (14)	0.0098 (15)	0.0093 (13)
N3	0.0346 (13)	0.0359 (11)	0.0312 (12)	0.0108 (10)	0.0107 (10)	0.0009 (10)
C1	0.0339 (14)	0.0323 (13)	0.0257 (13)	0.0064 (11)	0.0114 (11)	0.0070 (11)
C2	0.0279 (14)	0.0361 (13)	0.0307 (14)	0.0080 (11)	0.0096 (11)	0.0086 (12)
C3	0.0431 (16)	0.0291 (13)	0.0390 (16)	0.0075 (12)	0.0106 (13)	0.0055 (13)
C4	0.0427 (17)	0.0335 (14)	0.0363 (16)	0.0059 (12)	0.0076 (13)	−0.0035 (13)
C5	0.0383 (16)	0.0432 (15)	0.0241 (14)	0.0057 (12)	0.0069 (12)	0.0014 (12)
C6	0.0322 (15)	0.0402 (14)	0.0281 (14)	0.0067 (11)	0.0090 (12)	0.0086 (12)
C7	0.0400 (15)	0.0316 (12)	0.0281 (13)	0.0086 (11)	0.0109 (12)	0.0099 (11)
C8	0.0353 (15)	0.0329 (13)	0.0312 (14)	0.0086 (11)	0.0127 (12)	0.0090 (12)
C9	0.0388 (15)	0.0338 (12)	0.0196 (12)	0.0091 (11)	0.0065 (11)	0.0035 (11)
C10	0.0381 (16)	0.0380 (13)	0.0330 (15)	0.0132 (12)	0.0124 (12)	0.0049 (12)
C11	0.0368 (16)	0.0415 (14)	0.0351 (15)	0.0111 (12)	0.0047 (13)	0.0057 (13)
C12	0.0388 (16)	0.0370 (13)	0.0238 (13)	0.0114 (12)	0.0057 (12)	0.0090 (12)
C13	0.0366 (16)	0.0369 (13)	0.0257 (14)	0.0083 (12)	0.0099 (12)	0.0088 (12)
O4	0.0509 (15)	0.124 (2)	0.0492 (14)	0.0202 (14)	0.0114 (12)	0.0327 (16)
O5	0.0605 (14)	0.0674 (13)	0.0331 (11)	0.0222 (12)	0.0059 (11)	0.0138 (10)
C14	0.0446 (19)	0.0634 (18)	0.0416 (17)	0.0173 (15)	0.0120 (14)	0.0270 (15)
C15	0.0532 (19)	0.0627 (18)	0.0337 (16)	0.0231 (15)	0.0189 (14)	0.0227 (15)
C16	0.0535 (19)	0.0380 (14)	0.0285 (15)	0.0154 (13)	0.0083 (13)	0.0143 (13)
C17	0.0451 (17)	0.0528 (16)	0.0412 (17)	0.0194 (14)	0.0127 (14)	0.0219 (15)
C18	0.060 (2)	0.0560 (17)	0.0375 (17)	0.0286 (16)	0.0190 (15)	0.0192 (15)
C19	0.058 (2)	0.0485 (16)	0.0310 (15)	0.0176 (15)	0.0085 (14)	0.0141 (14)
O6	0.0598 (13)	0.0374 (10)	0.0392 (11)	0.0099 (9)	0.0066 (10)	0.0142 (9)
O7	0.0385 (12)	0.0613 (12)	0.0441 (12)	0.0148 (10)	0.0013 (10)	0.0015 (10)
O8	0.0590 (13)	0.0559 (12)	0.0278 (10)	0.0140 (10)	0.0012 (10)	0.0016 (10)
N4	0.0430 (13)	0.0301 (11)	0.0250 (11)	0.0077 (10)	0.0048 (10)	0.0043 (10)
N5	0.0626 (18)	0.0578 (16)	0.0342 (14)	0.0182 (14)	0.0108 (13)	0.0170 (14)
N6	0.0377 (14)	0.0402 (12)	0.0324 (13)	0.0130 (10)	0.0031 (11)	0.0013 (11)
C20	0.0294 (14)	0.0374 (13)	0.0269 (14)	0.0094 (11)	0.0063 (11)	0.0058 (12)
C21	0.0313 (15)	0.0440 (15)	0.0297 (14)	0.0094 (12)	0.0084 (12)	0.0075 (13)
C22	0.0388 (16)	0.0566 (17)	0.0276 (15)	0.0112 (14)	0.0074 (13)	0.0048 (14)
C23	0.0423 (17)	0.0411 (15)	0.0350 (16)	0.0042 (13)	0.0086 (14)	−0.0084 (13)
C24	0.0436 (17)	0.0355 (14)	0.0389 (16)	0.0108 (12)	0.0096 (14)	0.0029 (13)
C25	0.0329 (15)	0.0357 (13)	0.0278 (14)	0.0091 (11)	0.0086 (12)	0.0053 (12)
C26	0.0343 (15)	0.0317 (13)	0.0362 (15)	0.0082 (11)	0.0099 (12)	0.0114 (13)
C27	0.0375 (15)	0.0351 (13)	0.0325 (14)	0.0088 (12)	0.0086 (12)	0.0091 (12)
C28	0.0416 (16)	0.0311 (12)	0.0255 (13)	0.0098 (12)	0.0052 (12)	0.0058 (11)
C29	0.0325 (16)	0.0374 (14)	0.0280 (14)	0.0058 (12)	0.0043 (12)	0.0072 (12)
C30	0.0448 (17)	0.0328 (13)	0.0305 (15)	0.0068 (12)	0.0051 (13)	0.0087 (13)
C31	0.0360 (16)	0.0429 (15)	0.0395 (16)	0.0068 (12)	0.0040 (13)	0.0034 (13)
C32	0.0346 (15)	0.0412 (14)	0.0381 (16)	0.0100 (12)	0.0051 (13)	0.0061 (13)

### 3-(4-Amino-1-oxo-1,3-dihydro-2H-isoindol-2-yl)piperidine-2,6-dione–benzene-1,3-diol (2/1) Geometric parameters (Å, º)

**Table:**

O1—C8	1.230 (3)	C15—C16	1.371 (4)
O2—C12	1.220 (3)	C16—C17	1.384 (3)
O3—C13	1.209 (3)	C17—C18	1.384 (4)
N1—C8	1.367 (3)	C18—C19	1.371 (4)
N1—C9	1.437 (3)	O6—C26	1.242 (3)
N1—C7	1.460 (3)	O7—C29	1.202 (3)
N2—C6	1.370 (3)	O8—C30	1.225 (3)
N3—C12	1.368 (3)	N4—C26	1.358 (3)
N3—C13	1.379 (3)	N4—C28	1.444 (3)
C1—C2	1.378 (3)	N4—C27	1.458 (3)
C1—C6	1.390 (3)	N5—C21	1.392 (3)
C1—C7	1.497 (3)	N6—C30	1.370 (3)
C2—C3	1.385 (3)	N6—C29	1.373 (3)
C2—C8	1.477 (3)	C20—C25	1.371 (3)
C3—C4	1.386 (3)	C20—C21	1.396 (3)
C4—C5	1.375 (3)	C20—C27	1.494 (3)
C5—C6	1.397 (3)	C21—C22	1.389 (3)
C9—C10	1.519 (3)	C22—C23	1.390 (4)
C9—C13	1.522 (3)	C23—C24	1.382 (4)
C10—C11	1.514 (3)	C24—C25	1.391 (3)
C11—C12	1.493 (3)	C25—C26	1.470 (3)
O4—C14	1.375 (3)	C28—C29	1.519 (3)
O5—C16	1.373 (3)	C28—C32	1.519 (3)
C14—C19	1.377 (4)	C30—C31	1.482 (3)
C14—C15	1.384 (4)	C31—C32	1.513 (3)
			
C8—N1—C9	122.6 (2)	C15—C16—C17	121.2 (3)
C8—N1—C7	113.20 (19)	O5—C16—C17	121.4 (3)
C9—N1—C7	124.16 (18)	C16—C17—C18	118.2 (3)
C12—N3—C13	127.7 (2)	C19—C18—C17	121.7 (3)
C2—C1—C6	121.4 (2)	C18—C19—C14	118.7 (3)
C2—C1—C7	109.99 (19)	C26—N4—C28	122.7 (2)
C6—C1—C7	128.6 (2)	C26—N4—C27	112.5 (2)
C1—C2—C3	122.2 (2)	C28—N4—C27	124.77 (18)
C1—C2—C8	108.6 (2)	C30—N6—C29	127.0 (2)
C3—C2—C8	129.2 (2)	C25—C20—C21	121.0 (2)
C2—C3—C4	116.5 (2)	C25—C20—C27	110.0 (2)
C5—C4—C3	121.7 (2)	C21—C20—C27	129.0 (2)
C4—C5—C6	121.8 (2)	C22—C21—N5	122.4 (2)
N2—C6—C1	121.8 (2)	C22—C21—C20	116.5 (2)
N2—C6—C5	121.8 (2)	N5—C21—C20	121.1 (2)
C1—C6—C5	116.3 (2)	C21—C22—C23	121.6 (2)
N1—C7—C1	102.00 (18)	C24—C23—C22	122.0 (2)
O1—C8—N1	124.7 (2)	C23—C24—C25	115.6 (3)
O1—C8—C2	129.1 (2)	C20—C25—C24	123.2 (2)
N1—C8—C2	106.2 (2)	C20—C25—C26	108.2 (2)
N1—C9—C10	114.20 (18)	C24—C25—C26	128.6 (2)
N1—C9—C13	111.7 (2)	O6—C26—N4	123.9 (2)
C10—C9—C13	111.10 (19)	O6—C26—C25	129.0 (2)
C11—C10—C9	109.87 (19)	N4—C26—C25	107.1 (2)
C12—C11—C10	113.0 (2)	N4—C27—C20	102.16 (18)
O2—C12—N3	120.2 (2)	N4—C28—C29	112.0 (2)
O2—C12—C11	123.0 (2)	N4—C28—C32	114.32 (19)
N3—C12—C11	116.8 (2)	C29—C28—C32	109.95 (19)
O3—C13—N3	120.5 (2)	O7—C29—N6	120.8 (2)
O3—C13—C9	123.3 (2)	O7—C29—C28	124.4 (2)
N3—C13—C9	116.1 (2)	N6—C29—C28	114.8 (2)
O4—C14—C19	121.5 (3)	O8—C30—N6	119.8 (2)
O4—C14—C15	117.6 (2)	O8—C30—C31	122.6 (2)
C19—C14—C15	120.9 (3)	N6—C30—C31	117.6 (2)
C16—C15—C14	119.1 (2)	C30—C31—C32	114.8 (2)
C15—C16—O5	117.4 (2)	C31—C32—C28	109.7 (2)
			
C6—C1—C2—C3	0.0 (4)	O5—C16—C17—C18	−179.5 (2)
C7—C1—C2—C3	179.5 (2)	C16—C17—C18—C19	−0.2 (4)
C6—C1—C2—C8	−179.2 (2)	C17—C18—C19—C14	−1.6 (4)
C7—C1—C2—C8	0.4 (3)	O4—C14—C19—C18	−178.7 (2)
C1—C2—C3—C4	0.0 (4)	C15—C14—C19—C18	2.0 (4)
C8—C2—C3—C4	179.0 (2)	C25—C20—C21—C22	0.0 (3)
C2—C3—C4—C5	0.2 (4)	C27—C20—C21—C22	−178.8 (2)
C3—C4—C5—C6	−0.3 (4)	C25—C20—C21—N5	−177.9 (2)
C2—C1—C6—N2	179.4 (2)	C27—C20—C21—N5	3.3 (4)
C7—C1—C6—N2	−0.1 (4)	N5—C21—C22—C23	177.7 (2)
C2—C1—C6—C5	−0.1 (3)	C20—C21—C22—C23	−0.2 (4)
C7—C1—C6—C5	−179.6 (2)	C21—C22—C23—C24	−0.3 (4)
C4—C5—C6—N2	−179.2 (2)	C22—C23—C24—C25	0.9 (4)
C4—C5—C6—C1	0.3 (4)	C21—C20—C25—C24	0.6 (4)
C8—N1—C7—C1	0.1 (2)	C27—C20—C25—C24	179.7 (2)
C9—N1—C7—C1	−177.6 (2)	C21—C20—C25—C26	−179.5 (2)
C2—C1—C7—N1	−0.3 (2)	C27—C20—C25—C26	−0.4 (3)
C6—C1—C7—N1	179.2 (2)	C23—C24—C25—C20	−1.0 (4)
C9—N1—C8—O1	−1.6 (4)	C23—C24—C25—C26	179.1 (2)
C7—N1—C8—O1	−179.4 (2)	C28—N4—C26—O6	1.3 (4)
C9—N1—C8—C2	177.9 (2)	C27—N4—C26—O6	178.2 (2)
C7—N1—C8—C2	0.1 (3)	C28—N4—C26—C25	−178.7 (2)
C1—C2—C8—O1	179.2 (2)	C27—N4—C26—C25	−1.9 (3)
C3—C2—C8—O1	0.1 (4)	C20—C25—C26—O6	−178.7 (2)
C1—C2—C8—N1	−0.3 (3)	C24—C25—C26—O6	1.2 (4)
C3—C2—C8—N1	−179.4 (2)	C20—C25—C26—N4	1.4 (3)
C8—N1—C9—C10	−119.2 (2)	C24—C25—C26—N4	−178.7 (2)
C7—N1—C9—C10	58.3 (3)	C26—N4—C27—C20	1.6 (3)
C8—N1—C9—C13	113.7 (2)	C28—N4—C27—C20	178.4 (2)
C7—N1—C9—C13	−68.8 (3)	C25—C20—C27—N4	−0.7 (3)
N1—C9—C10—C11	177.73 (19)	C21—C20—C27—N4	178.3 (2)
C13—C9—C10—C11	−54.9 (3)	C26—N4—C28—C29	−124.1 (2)
C9—C10—C11—C12	53.1 (3)	C27—N4—C28—C29	59.5 (3)
C13—N3—C12—O2	179.7 (2)	C26—N4—C28—C32	110.0 (3)
C13—N3—C12—C11	0.6 (4)	C27—N4—C28—C32	−66.5 (3)
C10—C11—C12—O2	154.8 (2)	C30—N6—C29—O7	−176.8 (2)
C10—C11—C12—N3	−26.2 (3)	C30—N6—C29—C28	3.6 (3)
C12—N3—C13—O3	−179.5 (2)	N4—C28—C29—O7	13.1 (3)
C12—N3—C13—C9	−3.2 (3)	C32—C28—C29—O7	141.4 (2)
N1—C9—C13—O3	−24.3 (3)	N4—C28—C29—N6	−167.24 (19)
C10—C9—C13—O3	−153.0 (2)	C32—C28—C29—N6	−39.0 (3)
N1—C9—C13—N3	159.58 (19)	C29—N6—C30—O8	−169.0 (2)
C10—C9—C13—N3	30.8 (3)	C29—N6—C30—C31	11.9 (4)
O4—C14—C15—C16	−179.9 (2)	O8—C30—C31—C32	−168.9 (2)
C19—C14—C15—C16	−0.6 (4)	N6—C30—C31—C32	10.2 (3)
C14—C15—C16—O5	179.8 (2)	C30—C31—C32—C28	−44.5 (3)
C14—C15—C16—C17	−1.3 (4)	N4—C28—C32—C31	−174.74 (19)
C15—C16—C17—C18	1.6 (4)	C29—C28—C32—C31	58.3 (3)

### 3-(4-Amino-1-oxo-1,3-dihydro-2H-isoindol-2-yl)piperidine-2,6-dione–benzene-1,3-diol (2/1) Hydrogen-bond geometry (Å, º)

Ring centroids are defined as: *Cg*1 = N1/C7/C1/C2/C8; *Cg*2 = N3/C9–C13; *Cg*3 = C1–C6;*Cg*5 = N4/C20/C25–C27; *Cg*6 = N6/C28–C32; *Cg*7 = C20–C25; and *Cg*9 = C14–C19. *Cg*···*Cg* denotes the centroid—centroid distance associated with π–π interactions.

**Table:**

*D*—H···*A*	*D*—H	H···*A*	*D*···*A*	*D*—H···*A*
O4—H1···O8^i^	0.93 (3)	1.92 (3)	2.816 (3)	161 (3)
O5—H5*A*···O6	0.85 (3)	1.87 (3)	2.710 (3)	171 (3)
N2—H2*B*···O5^ii^	0.78 (3)	2.56 (3)	3.310 (3)	163 (3)
N2—H2*A*···O5^iii^	0.83 (3)	2.28 (3)	3.015 (3)	148 (3)
N3—H3···O1^iv^	0.89 (2)	2.31 (2)	3.022 (3)	137 (2)
N3—H3···O8	0.89 (2)	2.51 (2)	3.256 (3)	141 (2)
N5—H1*D*···O2^v^	0.92 (3)	2.23 (3)	3.144 (3)	172 (2)
N6—H6···O2	0.92 (3)	2.00 (3)	2.915 (3)	171 (2)
C9—H9···O3^iv^	0.98	2.65	3.409 (3)	135
C17—H17···O4^vi^	0.93	2.57	3.263 (3)	131
C27—H27*B*···O7	0.97	2.58	3.106 (3)	114
C31—H31*B*···O2^vii^	0.97	2.47	3.440 (3)	178
C10—H10*A*···Cg9^ii^		2.65	3.581 (3)	161
Cg1···Cg7^iii^			3.7109 (16)	
Cg1···Cg7^viii^			4.0014 (17)	
Cg3···Cg5^iii^			3.7997 (17)	
Cg3···Cg5^viii^			3.9603 (17)	
Cg3···Cg7^iii^			3.6971 (17)	
Cg3···Cg7^viii^			3.6644 (17)	
Cg3···Cg8^iii^			3.5854 (15)	
Cg3···Cg8^viii^			3.6382 (15)	
Cg4···Cg7^iii^			3.5410 (15)	
Cg4···Cg7^viii^			3.6602 (15)	
Cg4···Cg8^iii^			3.6763 (13)	
Cg4···Cg8^viii^			3.8723 (13)	

Symmetry codes: (i) −*x*+2, −*y*+1, −*z*+1; (ii) −*x*+1, −*y*+1, −*z*+1; (iii) *x*, *y*+1, *z*+1; (iv) −*x*+1, −*y*+2, −*z*+1; (v) −*x*, −*y*+1, −*z*; (vi) *x*−1, *y*, *z*; (vii) *x*+1, *y*, *z*; (viii) *x*+1, *y*+1, *z*+1.
